# MAFA: A Master Regulator of β-Cell Maturation and Function

**DOI:** 10.3390/cells15131199

**Published:** 2026-07-01

**Authors:** Lizabeth Johnson, Mallory A. Maurer, Jeeyeon Cha

**Affiliations:** 1 Department of Molecular Physiology and Biophysics, Vanderbilt University, Nashville, TN 37232, USA; lizabeth.buzzelli@vanderbilt.edu; 2 Division of Diabetes, Endocrinology, and Metabolism, Department of Medicine, Vanderbilt University Medical Center, Nashville, TN 37232, USA; m.a.maurer@vumc.org; 3 Geriatric Research Education and Clinical Center (GRECC) and Medicine Section, Veterans Affairs Tennessee Valley Healthcare System, Nashville, TN 37232, USA

**Keywords:** MAFA, beta cell, insulin, diabetes, pancreas

## Abstract

Dynamic insulin secretion and glucose homeostasis are dependent on the appropriate maturation and function of pancreatic β-cells. The islet-enriched musculoaponeurotic fibrosarcoma oncogene family A (MAFA) transcription factor acts as a master regulator of β-cell identity and function, coordinating gene expression networks required for glucose-stimulated insulin secretion. Dysregulation of MAFA contributes to β-cell dysfunction, as reduced expression is detected early in the pathogenesis of Type 1 Diabetes and Type 2 Diabetes, while long-lived variants can drive monogenic forms of diabetes. In this review, we summarize the current understanding of MAFA on β-cell maturation and function in both the mouse and human. This includes structural features of the MAFA protein, regulation of *MAFA* transcription, post-translational modifications, and emerging areas of research for therapeutic potential.

## 1. Introduction

As of 2024, 1 in 9 adults are living with diabetes worldwide, resulting in over 3 million deaths in 2024 and an estimated 1 trillion dollars in economic burden [1]. Diabetes mellitus is a heterogeneous collection of diseases characterized by eventual beta (β)-cell failure to regulate blood glucose levels through insulin secretion. β-cells reside in the pancreatic islets of Langerhans—small clusters of endocrine cells within the pancreas—and are the only cells in the body to secrete insulin in response to glucose. β-cells coordinate with other islet cell types, including glucagon-secreting α-cells and somatostatin-secreting δ-cells, to ultimately regulate systemic glucose levels. β-cell failure can result from a range of insults, including autoimmune destruction (Type 1 Diabetes; T1D), relative insulin deficiency driven by chronic metabolic stress and insulin resistance (Type 2 Diabetes; T2D), stressors during pregnancy (Gestational Diabetes), hereditary mutations that impair β-cell function (Monogenic Diabetes), and diabetogenic viral infections [2,3] and exposure to β-cell toxins, including immunosuppressive drugs [4].

Several islet-enriched transcription factors (TFs) have been identified as critical regulators of gene expression to promote β-cell identity and function. Among these, musculoaponeurotic fibrosarcoma oncogene family A (MAFA) is uniquely enriched in pancreatic islet β-cells and is a critical regulator of β-cell identity and maturation [5,6,7,8,9,10,11]. MAF TFs were first identified as cellular proto-oncogene counterparts of the avian musculoaponeurotic fibrosarcoma virus AS42 in the late 1980s [12,13]. Since then, 7 MAF family TFs have been identified and characterized based on size: the large MAFs (c-MAF, MAFA, MAFB, NRL) and the small MAFs (MAFF, MAFG, MAFK) [14,15]. These TFs contribute to the development, differentiation, and maintenance of a diverse range of cellular functions in many organs and tissues, such as in the lens [16,17] and pancreatic islet [18]. 3 MAF TFs (c-MAF, MAFA, and MAFB) are detected within the islet and can bind to and activate *Insulin* (*Ins1/2*, mouse) and *INSULIN* (*INS*, human) expression [18].

Previous reviews have established MAFA as a critical regulator of β-cell maturation and *Insulin* gene transcription [7,8,9,10,11]. Here, we review the structure, expression, and function of MAFA in β-cells under physiologic and metabolic stress conditions. We further highlight recent advances that have substantially expanded our understanding of MAFA biology. These include the identification of *MAFA* as a causative monogenic Maturity-Onset Diabetes of the Young (MODY) gene, emerging roles in circadian timing of MAFA activity, insights into the functional relevance of MAFA structural elements predicted by AlphaFold, and growing evidence that MAFA is a key determinant of stem-cell-derived (SC)-β-cell maturation. By integrating findings from mouse and human islet studies, stem-cell models, and natural history of monogenic disease, we highlight how MAFA functions as a dynamic regulator of β-cell identity and glucose homeostasis across developmental, physiological, and disease contexts. We also discuss major unresolved questions regarding MAFA regulation and function that may inform future therapeutic strategies for diabetes and β-cell replacement therapies.

## 2. Protein Structure and Functional Domains of MAFA

**Figure 1:**
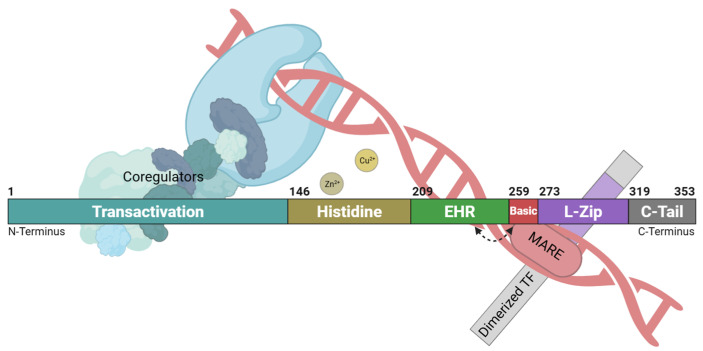
Musculoaponeurotic fibrosarcoma oncogene family A (MAFA) protein structural elements work together to coordinate MAFA biology. The MAFA protein is 353 amino acids in length and comprises six domains with distinct functions: a transcriptional activation domain (Transactivation), a histidine-rich domain (Histidine), an extended homology region (EHR), a basic region (Basic), a leucine-zipper (L-Zip), and a C-terminal tail (C-Tail). The transactivation domain is involved in binding coregulators that influence gene transcription, while the histidine-rich domain, EHR, and basic region work together to bind MAFA to DNA via MAF recognition elements (MAREs). The EHR does not directly interact with MARE sequences but instead stabilizes DNA binding by interacting with the basic domain, as indicated with an arrow. The leucine zipper allows dimerization with another MAFA protein or other transcription factor (TF), which impacts the transcriptional activity. Figure made with BioRender.

**Figure 2:**
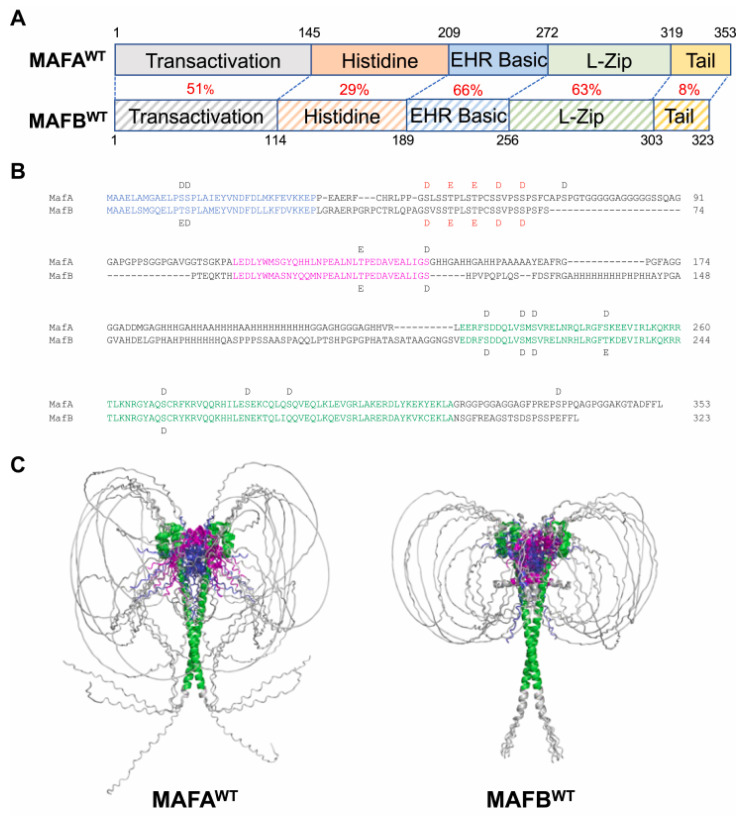
Protein sequence and AlphaFold 2 modeling of MAFA^wild-type (WT)^ and MAF family B (MAFB^WT^) proteins. (**A**) protein sequence identities (%) between various domains of MAFA^WT^ and MAFB^WT^ are denoted in red. Extended homology domain (EHR); Leucine-zipper (L-Zip). (**B**) The aligned amino acid sequences of MAFA^WT^ and MAFB^WT^, with aspartic acid (D) and glutamic acid (E) used as phosphomimics at bona fide sites of phosphorylation [20]. The red-labeled E and D represent the phosphosites absent in the underphosphorylated variants MAFA^S64F^ and MAFB^S70A^. The regions marked in blue (i.e., 83%) and magenta (i.e., 79%) have higher sequence conservation than the overall transactivation domain and have structural elements in the AlphaFold 2 models. The region in green is the basic-leucine-zipper domain structured by AlphaFold 2 and determined by crystallography (i.e., MAFA residues 226–318 and MAFB residues 211–302) [22,39]. (**C**) Comparing the five independently derived representative models for MAFA^WT^ (left) and MAFB^WT^ (right) illustrates a potential structural difference. For example, the longer C-terminal tail of MAFA^WT^ leads to a more divergent conformation than the MAFB^WT^, which may interact with N-terminal region sequences. These structural differences may contribute to the distinct transcriptional activities of MAFA and MAFB in β-cells despite their shared DNA-binding domain. Adapted from Cha et al., 2024 [19].

MAFA is 353 amino acids (aa) in length and includes several structural domains (Figure 1), including a coregulatory binding domain (N-terminus transcriptional activation domain [TAD; aa1–145]), DNA-binding domains (a histidine-rich region [aa146–208], an extended homology region [EHR; aa209–258], a basic region [aa259–272]), a dimerization region (leucine-zipper [aa273–318]) and a C-terminal tail [aa319–353] [19,20]. The highest sequence variability between MAFA and other large MAF proteins lies within the C-terminal tail and histidine-rich regions, whereas the EHR and basic-leucine zipper (bZIP) domains are highly conserved (Figure 2A) [19].

### 2.1. Transactivation Domain (TAD)

A defining feature of large MAF proteins is the presence of the TAD, which enables MAF dimers to bind to coregulatory proteins. Coregulators influence MAFA-regulated gene expression by participating in the recruitment of transcriptional machinery and/or chromatin modifiers such as islet-enriched TFs, methyl transferases/de-transferases, coactivators, and scaffolding proteins [21] (See Section 5).

### 2.2. DNA-Binding Domains

The enhanced (extended) homology region (EHR), basic domain, and histidine-rich region coordinate MAFA DNA-binding specificity and transcriptional activity. Large MAF proteins utilize the EHR domains to enhance binding to 13–14 base pair DNA motifs known as MAF recognition elements (MAREs) in target genes [22,23,24]. There are two types of MARE sequences: a 13-base pair (bp) T-MARE sequence and a 14-bp C-MARE sequence. Within each of these sequences, a 7-bp TPA-response element (TRE; 5′-TGA(G/C)TCA-3′) or an 8-bp cyclic AMP-response element (CRE; 5′-TGACGTCA-3′) is at the core of the T-MARE and C-MARE sequences, respectively, both flanked by extended GC-rich sequences (5′-TGC-(TRE/CRE)-GCA-3′) [23,24,25].

MAFA interaction with MARE sequences is phosphorylation-dependent [20,26] (See Section 4.1). The basic domain directly binds the core regions of target MARE sequences [25]. The EHR does not directly interact with MARE sequences but instead stabilizes DNA binding by interacting with the basic domain [22]. Inference from other basic leucine-zipper (bZIP) TFs suggests that this interaction induces a conformational change in the basic domain α-helix from a flexible to a structured state to stabilize DNA binding [27].

Histidine-rich regions are known to affect protein stability and DNA binding [28,29,30], and can readily interact with Cu^2+^ and Zn^2+^ metal ions [31]. How these metal-ion interactions coordinate specific MAFA coregulator recruitment or DNA-binding specificity is not well described and will require structural and proteomic approaches to identify metal-dependent cofactors and target-site selection mechanisms.

### 2.3. Leucine-Zipper Domain

The leucine-zipper domain is a leucine-rich coiled region that facilitates dimerization through hydrophobic interactions. MAFA imparts transcriptional activity as a dimer and can form either homodimers or heterodimers to work synergistically with other TFs. Such interactions have been characterized in a variety of cell types for MAF family proteins, including dimerization with other MAFs, BACH2 [32], NRF2 [33], FOS and JUN [24,34,35] to increase transactivation [36]. This domain also coordinates with other MAFA domains by modulating interactions with phosphorylation sites in the N-terminus, affecting DNA-binding and MAFA stability [20,35,37,38].

### 2.4. C-Terminal Tail

There have been limited studies investigating the direct role of the C-terminal tail of MAFA. A recent study using AlphaFold 2 structural predictions of MAFA homodimers has facilitated understanding of this disordered region and shows the non-conserved C-terminal tail likely holds a secondary structure that lies near the DNA-binding domain (Figure 2C). Functional assessment of chimeric proteins with C-terminal tails interchanged between MAFA and MAFB reveals that the C-terminal tail is important for cooperativity with other islet-enriched TFs in target gene activation, independent of DNA binding [19].

## 3. Islet MAFA Expression During Development and Adulthood

While the expression and regulation of other islet-enriched TFs are similar between humans and rodents [40,41,42,43], there are distinct species-specific expression patterns for human *MAFA* and mouse *MafA*. Rodent models continue to provide essential fundamental mechanistic insight into MAFA biology, but considering species-specific differences is essential for improving the translational relevance of preclinical studies. These key differences are summarized in Table 1.

### 3.1. Rodent Models

In mice, pancreatic development is characterized by two sequential stages: the primary wave, lasting until embryonic day (E) 13.5, and the secondary wave, lasting until birth (E18.5). Organ determination is established during the primary wave, and initial pancreas formation is observed by E9.5 [50,56]. By E9.5, primary β-cell specification begins with multipotent progenitor cell differentiation within the pancreatic epithelium. Glucagon-producing cells appear first, followed by insulin-producing cells at approximately E10.5 [57,58]. During this primary wave of development, these hormone-producing cells exist in a “protodifferentiated state”, in which they produce minimal amounts of hormone and are unresponsive to glucose stimulation due to a lack of key glucose uptake proteins [59]. *MafA* expression is induced during the transition to the secondary wave (E12.5–E13.5) exclusively in insulin-producing cells [6,44].

**Figure 3:**
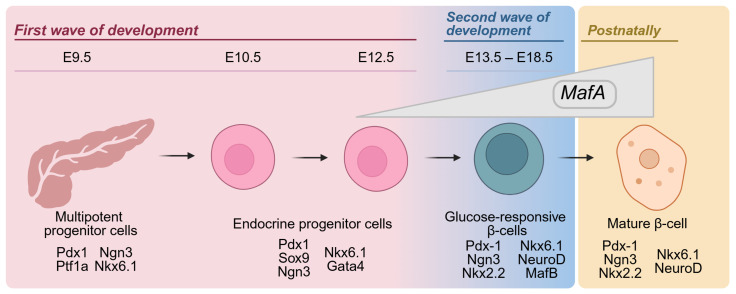
*MafA* expression drives β-cell maturity during development, characterized by glucose-regulated insulin secretion in mice. This schematic summarizes the timing of *MafA* expression and other TFs governing the development and differentiation of mouse β-cells. The pink section depicts the first wave of development from embryonic day (E)9.5, the beginning of pancreatic formation and multipotent progenitor cells, to the first time point *MafA* is detectable in insulin-producing cells. The blue section displays the second wave of development from E13.5 to birth (E18.5), characterized by increased protein synthesis driven by MafA, resulting in glucose-responsive β-cells. The yellow section illustrates the postnatal result of the mature β-cell with high *MafA* expression. Figure made with BioRender.

The secondary wave is characterized by a significant increase in protein synthesis [60]. *MafA* expression drives acquisition of the glucose-responsive, mature β-cell phenotype, in part through induction of *Slc2a2* (encoding Glut2) and *Ins1/2* expression [9,61,62]. Most pancreatic β-cells are generated during this differentiation wave through the contribution of many transcriptional regulators in conjunction with MafA (Figure 3). *MafA* remains highly expressed in this period and is the dominant Maf factor in β-cells postnatally throughout adulthood [44,45].

### 3.2. Human Models

Human *MAFA* expression is undetectable within the pancreas until the second trimester and remains low during early postnatal life in β-cells until approximately 9 years of age [46,47,48]. Similarly, postnatal MAFA protein levels are rarely detected in juvenile human β-cells until approximately 10 years of age [48]. In contrast, rodent *MafA* expression is induced during embryonic development and becomes the dominant Maf TF in mature β-cells shortly after birth (Figure 3) [44].

To better understand this discrepancy, investigators have leveraged collaborative large-scale initiatives to acquire human pancreata, such as the Network for Pancreatic Organ Donors with Diabetes (nPOD), Human Pancreas Analysis Program (HPAP), and the Human Islet Research Network (HIRN) [63,64,65]. These efforts have enhanced our understanding of human *MAFA* expression through integrated transcriptomic, epigenomic, and single-cell analyses of healthy and diabetic islets. These datasets support a model in which *MAFA* expression is associated with mature β-cell states [51] and is diminished in dysfunctional β-cells from individuals with diabetes [52].

Human single-cell transcriptomic studies identify *MAFA* expression as one of the most reliable markers of β-cell identity, with expression correlating with improved β-cell function [51]. Adult human β-cells frequently co-express *MAFA* and another MAF TF, *MAFB*, unlike rodents, in which *MafB* expression is largely restricted to α-cells [45]. Human β-cells co-expressing *MAFA* and *MAFB* display enriched expression of genes involved in insulin secretion, mitochondrial metabolism, and β-cell function [51]. This suggests species-specific MAFA transcriptional networks within the β-cell. Transcriptionally, studies in both the human β-cell line EndoC-βH1 and human islets demonstrate MAFA as a regulator of exocytotic machinery [66]. Functionally, loss of *MAFA* impairs both glucose- and K^+^- stimulated insulin secretion in EndoC-βH1 cells [66]. Moreover, rodent MafA is required for the maintenance of β-cell identity and repression of non-β-cell hormones such as glucagon, somatostatin, and gastrin [67]. However, this role is fulfilled by MAFB in human β-cells [67]. These studies indicate that while MAFA serves a conserved role in promoting β-cell maturity across species, the timing of expression, interactions with other TFs, and pathways dysregulated in the absence of these factors differ substantially between rodents and humans.

Human embryonic stem (hES) cells are capable of directed differentiation to generate pancreatic and endocrine precursor cells, and ultimately insulin-secreting cells [68,69,70,71,72,73,74,75,76,77]. In these SC-β-cells, glucose-responsiveness is correlated with sustained *MAFA* expression [71,78]. Although hES cell models can provide insight into MAFA function in a human context, defining *MAFA* expression patterns during differentiation does not fully recapitulate the human condition. Continued integration of data from human islet-derived datasets and stem-cell models will be important for defining pathways and strategies regulating *MAFA* expression and activity in human β-cells [63,64,65] (See Section 7).

## 4. Regulation of MAFA Activity via Post-Translational Modifications

### 4.1. Phosphorylation

Blood glucose concentrations are regulated by dynamic insulin secretion from β-cells and precise regulation of MAFA via post-translational modifications (PTMs). Among these PTMs, the phosphorylation status of MAFA dictates many aspects of MAFA function, including dimerization [20], DNA binding [20,26,38], coregulator interactions [79], and ubiquitin-targeting for protein turnover [80]. Specifically, phosphorylation within the MAFA N-terminus is required for C-terminus dimerization with other proteins and coactivators [81]. Chemical dephosphorylation of MAFA by treatment with calf intestinal alkaline phosphatase reduced DNA-binding activity. These effects were largely prevented with the phosphatase inhibitor Na_3_VO_4_, suggesting that phosphorylation of MAFA positively impacts DNA binding [20,26].

Phosphorylation in the TAD has a dual role in regulating human MAFA activity, as it promotes both transcriptional activation and protein turnover. A mass spectrometry study revealed 10 phosphorylation sites within the MAFA TAD, 7 in the bZIP domain, and 1 in the C-terminus [20]. Of these sites, MAFA biological functions depend on the priming phosphorylation event of the TAD residue serine (Ser [S]) 65 [80]. Although the kinase responsible for this priming phosphorylation event is still unknown, phosphorylation of Ser65 triggers the sequential phosphorylation of TAD residues: S61, threonine (Thr [T]) 57, T53, and S49 by GSK-3 [81,82]. Phosphorylation of S14 is distinct from the GSK-3 cascade and not required for protein turnover, but instead is required for MAFA transcriptional activity [38,80,81,82].

### 4.2. Ubiquitination

Under low-glucose conditions, MAFA is rapidly marked for degradation by polyubiquitination [82]. Ubiquitination is a PTM in which the small 76 amino acid protein ubiquitin is conjugated onto a protein [83]. Following the key phosphorylation event at S65, MAFA is polyubiquitinated in the C-terminus and targeted for proteasomal degradation [80]. By a cycloheximide chase experiment, it was determined in vitro that MAFA has rapid protein turnover with a t_1/2_ of ~30 min [54] under low-glucose conditions. In contrast, under high-glucose conditions, the TAD residues are phosphorylated, and subsequent acetylation transiently prevents MAFA degradation, thereby promoting transcriptional activity [81,82]. Following transcription initiation, these same phosphorylated sites serve as signals for polyubiquitin via the E3 ligase HRD1 and eventual degradation [81,84].

### 4.3. Acetylation

PCAF (Lysine acetyltransferase 2b, or KAT2B) acetylates MAFA and protects it from ubiquitin-tagged degradation [81], although specific lysine residues or deacetylases involved have not yet been identified. Consistently, *KAT2b* (encoding PCAF) expression is decreased in both *db/db* mice and T2D human islets [85]. Disruption of *Kat2b* in mice also results in increased MAFA proteasomal degradation [86], although it has minimal impact on *MafA* mRNA levels [85]. Functionally, loss of PCAF protein impairs insulin secretion and results in overall glucose intolerance in mice from a loss of β-cell adaptation to stress [85]. Although the importance of acetylation in MAFA regulation of β-cell function is evident, future studies are needed to define signaling pathways that control PCAF activity and determine how altered MAFA acetylation contributes to β-cell dysfunction during diabetes pathogenesis.

### 4.4. SUMOylation

SUMOylation is a series of enzymatic reactions that covalently conjugate small ubiquitin-related modifiers (SUMOs) to lysine residues of target proteins. These PTMs are dynamic and readily removed by SUMO-specific proteases. MAFA can be post-translationally modified by two SUMO isoforms, SUMO-1 and SUMO-2, on Lys32, resulting in decreased transcriptional activity [87]. SUMOylation is known to regulate a wide range of processes in different cell types and tissues, including DNA repair, cell cycle, and gene transcription [83,88]. However, its exact influence on MAFA activity and protein levels within the β-cell requires examination of relevant SUMO ligases, proteases, and downstream transcriptional consequences.

## 5. Transcriptional Regulation of β-Cell Function by MAFA

β-cell maturation is defined by the ability to secrete insulin in response to glucose and the acquisition of β-cell-enriched identity genes, including *MAFA* [89,90,91]. Loss of *MafA* in the mouse pancreas [45,49], islet [5], or specifically in the β-cell [61] similarly results in altered β-cell identity and function. This includes impaired glucose-stimulated insulin secretion (GSIS) and transdifferentiation into other islet cell types. For example, β-cell-specific deletion of *MafA* under stress conditions induces inappropriate gastrin hormone production in mouse islets [67]. Notably, complete loss of rodent *MafA* is not required for dysfunction; hyperglycemia alone impairs MafA nuclear translocation and promotes progressive loss of β-cell identity [52,53,92].

The first identified MafA regulatory target in the pancreatic β-cell was the rat insulin promoter element 3b (RIPE3b) via the C1 element within the RIPE3b promoter [26,93,94]. This led to the original identification of MAFA as the RIPE3b activator [94]. MAFA has since been shown to regulate the expression of several genes involved in β-cell transcriptional maturation and functional competence in humans and mouse models, as summarized in Table 2.

The establishment and maintenance of β-cell GSIS requires complex coordination of transcriptional networks driven by islet-enriched factors such as MAFA, PDX1, NGN3, and NEUROD1 [108,109,110]. MAFA functions cooperatively with other TFs through heterodimerization and coregulator interactions mediated by its TAD (See Section 2.3), including other large MAF bZIP TFs such as MAFB and c-MAF [22] within the islet. β-cells co-expressing MAFA and another family member, MAFB, are functionally and transcriptionally more active than β-cells expressing MAFA homodimers alone [51], suggesting unique interactions with coregulatory proteins. MAFA also participates in multi-protein transcriptional complexes to regulate gene expression. Notably, MAFA acts synergistically with PDX1 and NEUROD1, although MAFA can independently activate *INS* expression [110].

In addition, MAFA recruits chromatin-modifying coactivators to facilitate the recruitment and assembly of RNA polymerase II and transcriptional machinery at target promoters. The mixed-lineage leukemia 3 (MLL3) and mixed-lineage leukemia 4 (MLL4) complexes, which catalyze enhancer-associated mono-methylation of histone 3 lysine residue 4 (H3K4me1), contribute to MAFA-dependent gene regulation [21]. Loss of β-cell-specific *Ncoa6* in mice, a core subunit of these complexes, reduces expression of target genes such as *Ins2* and *Slc2a2*, and impairs GSIS [21]. Similarly, histone acetyltransferases, including p300, CREB binding protein (CBP), and PCAF, can bind to Maf proteins [111], and are required for glucose-responsive, MAFA-mediated gene transcription [112]. Together, these findings highlight the importance of chromatin remodeling in enabling robust MAFA-mediated transcriptional programs to regulate the expression of downstream targets.

## 6. MAFA in Diabetes Pathogenesis

### 6.1. Type 1 Diabetes (T1D)

T1D is characterized by a tissue-specific autoimmune attack against insulin-producing β-cells, leading to an inability to appropriately regulate blood glucose levels [113]. Insulin is the dominant autoantigen in T1D [113], and reduced thymic *Insulin* expression is associated with increased insulin autoantibody production [114,115]. MafA is the only insulin-promoting TF identified thus far in the mouse thymus [116,117]. Global MafA knockout (KO) in mice decreased *Ins2* expression in the thymus and increased β-cell autoantibodies in serum [116,117]. NOD mice, a model of T1D, also show decreased *MafA* and *Ins2* thymic expression compared to control mice [117]. Genetic variants of MAFA affecting its expression or activity can influence T1D susceptibility in both mice and humans. *MafA* variants decreasing its promoter activity were identified exclusively in NOD mice compared to control and T2D mouse models [116]. Conversely, a single nucleotide polymorphism (Gly346Cys, rs6252187) increasing thymic MAFA transcriptional activity is correlated with a decreased risk of T1D in humans [118]. These results suggest that thymic *MAFA* expression or activity can potentially influence T1D susceptibility in mice and humans.

The immune microenvironment of the islet also plays a role in the pathogenesis of T1D. The expression of pro-inflammatory molecules in healthy islets is tightly regulated to avoid inappropriate immune activation. Decreased *MAFA* expression in human islets strongly correlated with cytokine-induced signaling and T1D susceptibility genes. Loss of *MafA* in mouse islets similarly resulted in an increase in pro-inflammatory genes in isolated islets, improved viral clearance compared to control islets [119], and increased accumulation of immune cells [120]. This suggests that islet *MafA* expression may also be critical for preventing autoimmune attack in T1D. However, an underlying mechanism for these changes remains unclear, and more research is needed to identify a causative link to T1D pathogenesis.

### 6.2. Type 2 Diabetes (T2D)

T2D is a chronic condition where β-cells fail to produce sufficient insulin alongside peripheral insulin resistance, resulting in high blood glucose levels. Both *MAFA* mRNA and MAFA protein levels are reduced in islets from T2D patients compared to non-diabetic controls [52,53,119]. Similarly, *MafA* expression is reduced in *db/db* mice, a model for T2D, obesity, and insulin resistance [121]. Restoring pancreas-specific *MafA* expression in mice improves β-cell mass, enhances function, and lowers blood glucose levels [122]. Notably, MAFA is susceptible to post-translational oxidative stress, demonstrating a 97% decrease in protein levels in response to glucotoxicity [123]. Chronic hyperglycemia creates glucolipotoxic conditions, which overwhelm β-cell mitochondria to accumulate reactive oxygen species (ROS). This oxidative stress increases MAFA turnover, decreases *MAFA* expression, and promotes MAFA mislocation to the cytoplasm, collectively reducing binding to the *Insulin* promoter [52,124]. Consequently, this impairs *Insulin* expression and GSIS [123,125], which can be prevented by treatment with antioxidants in vitro in a dose-dependent manner [123].

Given that *MAFA* is downregulated in islets from T2D donors and plays a pivotal role in regulating GSIS, *MAFA* overexpression has been explored as a potential therapeutic target in restoring β-cell function. *MAFA* overexpression alone or in combination with other islet-enriched TF’s can drive non-β-cells into functional insulin-secreting cells in vitro [126,127,128]. However, these approaches have remained unable to fully rescue diabetic phenotypes in vivo [129]. This discrepancy may reflect the exceptionally tight regulation of MAFA, where sustained overexpression is insufficiently maintained by autoregulation, likely via region 3 of the *MAFA* promoter [130]. These findings highlight the need for improved characterization of MAFA production and strategies that recapitulate precise endogenous *MAFA* regulation to achieve therapeutic benefit for T2D.

### 6.3. Maturity-Onset Diabetes of the Young (MODY)

Two missense mutations within the TAD of MAFA—Ser64Phe [54] (S64F) and Thr57Arg [131] (T57R)—have been identified to drive a form of monogenic diabetes called MODY diabetes [132]. Typical clinical characteristics of MODYs include noninsulin-dependent diabetes and early-onset, typically before the age of 25 years [132,133]. MAFA-MODY differs phenotypically with clinical presentation at age ~40 and presents in a sex-dependent manner. Female carriers are more likely to develop insulinomas while male carriers develop MODY, though this bias appears stronger with the Ser64Phe mutation.

These clinical phenotypes are partially recapitulated in a mouse model: male mutant mice harboring the Ser64Phe mutation develop diabetes, whereas female mutants are persistently hypoglycemic. Furthermore, sex-biased signatures of MafA^S64F^ point to downstream disruption of calcium dynamics in females versus early onset senescence and dysregulated circadian rhythm in males [55,107]. Although ovariectomy studies in mice have demonstrated that these differences are not principally due to postnatal estrogen [55], underlying etiology for these diametrically opposed phenotypes remains to be explored.

At the protein level, Ser64Phe mimics the effects of the kinase-priming defective mutation, Ser65Ala, suggesting that Ser64Phe disrupts the phosphorylation at Ser65 (See Section 4.1). As a result, MAFA^S64F^ has both increased protein stability and increased transactivation capacity. This disrupts the requisite and precise regulation of *MAFA* and downstream target regulation by MAFA, ultimately leading to impaired GSIS [54]. Similarly, Thr57 lies within the TAD and is recognized to be one of the subsequent phosphorylation events following the Ser65 phosphorylation. Therefore, while the mechanisms behind Thr57Arg have not been thoroughly studied, it is proposed to have similar functional consequences as Ser64Phe, including enhanced stability [131]. These familial cases of MAFA-MODY highlight the importance of MAFA phosphorylation and appropriate turnover on cellular function, supporting a model in which MAFA activity must be maintained within a functional range.

## 7. Emerging Roles and Novel Studies

### 7.1. Roles of MAFA in Glucose Homeostasis Beyond the Pancreatic β-Cell

While *MAFA* expression is enriched in the pancreatic islet, it is also highly expressed in other tissues, including skeletal muscle, adipose, testes, and regions of the brain [134,135]. Among these, its role in the skeletal muscle is well characterized. Skeletal muscle is a major contributor to whole-body glucose homeostasis, accounting for the majority of insulin-stimulated glucose uptake and serving as a key site of insulin resistance in diabetes [136]. Prolonged skeletal muscle inactivity, such as that which occurs in neuromuscular disease, induces a shift from oxidative type I myofibers to more glycolytic type II myofibers [137]. *MafA* is expressed in a subset of fetal myofibers in lambs, where it promotes maturation through activation of myofiber-specific maturity programs [138]. MafA also functions as a determinant of myofiber subtype specification in mice, specifically promoting type IIb myofiber identity during development and adaptation [139]. Since myofiber composition influences glucose utilization and metabolic flexibility [140], these findings position MAFA as a regulator of metabolic function in skeletal muscle. Modulation of MAFA activity may represent novel therapeutic avenues for insulin resistance related to muscular dystrophy.

In contrast, the roles of MAFA in other metabolic tissues remain less defined. In adipose tissue, loss of rodent *MafA* is associated with reduced expression of genes required for mature adipocyte differentiation, as well as alterations in cell growth and lipid droplet accumulation [141,142]. MAFA is also highly enriched in the testes, where it may influence male sex hormone production, although its functional role in this context remains unclear. Notably, nuclear androgen signaling can regulate islet function [143], raising the possibility of indirect crosstalk between MAFA activity in reproductive tissues and β-cell physiology. Consistent with this idea, the pronounced sex-specific phenotypes observed clinically in patients with mutations in MAFA (MAFA^S64F^ and MAFA^T57R^) suggest that MAFA-dependent pathways may contribute to differential metabolic vulnerability between males and females [144] (See Section 6.3). Investigation of sex-biased differences in expression and activity of MAFA regulators or MAFA variants is warranted.

*MAFA* expression has also been reported in discrete regions of the hypothalamus, including the ventromedial nucleus (VMH), arcuate nucleus (ARC), paraventricular nucleus (PVN), and lateral hypothalamus (LH), which are key regulators of energy balance and glycemic control [145]. Loss of *MafA* in the mouse brain is associated with downregulation of genes involved in food consumption and metabolism, including growth hormone, pro-melanin-concentrating hormone (MCH), and orexin [142]. These findings suggest that MAFA may also contribute to central regulation of energy homeostasis, although direct functional studies in specific neuronal populations are limited.

Taken together, emerging evidence suggests that MAFA may function beyond the β-cell to integrate tissue-specific aspects of metabolism across multiple organ systems. Addressing this question will require tissue-specific genetic models and metabolic phenotyping to determine how MAFA functions across organs are coordinated.

### 7.2. Circadian Regulation of MAFA

Circadian regulation of whole-body metabolism was first described over 50 years ago, when studies demonstrated diurnal variation in β-cell insulin secretion in humans [146,147,148]. Epidemiological studies have since shown that circadian misalignment, such as in night shift workers, is associated with a 60% increase in the development of diabetes [149,150]. Conversely, T2D patients exhibit blunted rhythmic insulin in response to mixed meals [151], suggesting that disruption of circadian control contributes to impaired insulin secretion. Together, these findings indicate that β-cell insulin secretory machinery is intrinsically tuned with circadian timing.

The circadian system is driven by transcriptional-translational feedback loops present in nearly all cell types, including pancreatic β-cells. These molecular clocks coordinate environmental cues, such as the light-dark cycles and feeding cycles, with cellular function, including cyclic insulin secretion. In β-cells, intrinsic autonomous oscillatory transcriptional cycles enable islets to anticipate nutrient availability and be primed for insulin demand [152]. The detailed molecular aspects of the circadian clock of the pancreatic β-cell have been extensively reviewed elsewhere [153,154,155]. Briefly, circadian activators, Circadian Locomotor Output Cycles Kaput (CLOCK) and Brain and Muscle ARNT-Like 1 (BMAL1), drive expression of clock-controlled genes, including circadian repressors *Period* (encoding PER1, PER2, and PER3) and *Cryptochrome* (encoding CRY1 and CRY2), as well as additional modifiers such as *Basic Helix-Loop-Helix Family Member E40 *(*BHLHE40*; encodes DEC1). These proteins form autoregulatory feedback loops that generate oscillations in gene expression and cellular function.

Recent studies have linked circadian entrainment to postnatal β-cell maturation. Establishment and maintenance of circadian rhythmicity are critical for β-cell maturity [156,157,158], which includes the induction of key maturation factors such as *MAFA*. Circadian entrainment promotes human islet maturation through chromatin remodeling, increasing accessibility at loci associated with insulin secretion, including *MAFA*, and enhancing GSIS responsiveness, although the specific chromatin modifiers remain unclear [157]. In differentiating hES cells, acquisition of glucose responsiveness coincides with chromatin remodeling events that enable access to both insulin-secretory genes and circadian regulators such as CLOCK and BMAL1 [157]. Notably, *MafA* is one of the only essential β-cell TFs that exhibits rhythmic expression in mice [159]. Disruption of the circadian regulator DEC1 in human SC-islets abolishes *MAFA* oscillatory expression and impairs GSIS [75]. Conversely, mouse models expressing stabilized MafA^S64F^ show impaired glucose tolerance with downregulated islet expression of circadian genes, including *Bhlhe40* [107], suggesting the existence of a conserved feedback relationship between MAFA and circadian regulators in mice and humans. Dec1 is also suggested to mediate hypoxia-induced β-cell function through suppression of *MafA* in mice [160]. The downregulation of *MafA* compromises ATP generation and exocytotic machinery, resulting in impaired β-cell GSIS [160].

Together, these findings suggest a bidirectional relationship between circadian regulation and β-cell transcriptional networks, in which MAFA both responds to and influences circadian timing mechanisms. Understanding how essential β-cell-specific TFs such as MAFA integrate circadian timing with specialized insulin-secretory programs will require integrating circadian perturbation, chromatin accessibility, transcriptomic profiling, and MAFA occupancy analysis in mature human β-cells. However, these results reveal that optimal β-cell function may require not only maintenance of appropriate MAFA levels but also cyclical periods of MAFA activity.

### 7.3. Stem Cell (SC)-Derived Therapies

Current therapies for diabetes effectively manage hyperglycemia but do not cure the disease. Standard treatment includes glucose monitoring and exogenous insulin administration, which requires frequent monitoring and dose adjustments, which increases risk for adverse events such as hypoglycemia. As a result, there is a need for alternative therapies to improve the overall treatment of diabetes while minimizing adverse outcomes. Transplantation of β-cells provides an attractive therapeutic approach, as these cells can sense glucose and secrete insulin in real-time. Cadaveric β-cells are often limited and difficult to acquire, while SC-β-cells offer a renewable source of β-cells, which may improve access to treatment [161].

Recent advances in SC-β-cell therapies have demonstrated SC-β-cell potential for clinical translation. As of 2025, Vertex Pharmaceuticals has completed Phase 1/2 clinical trials of an SC-derived islet transplantation therapy (Zimislecel) in individuals with T1D. One year after treatment, all 12 participants who received a full dose exhibited elimination of hypoglycemic events, improved glycemic control (HbA1c < 7%), and increased time in the target glucose range (70–180 mg/dL), with the majority achieving insulin independence thus far [162]. However promising, these pilot studies require longitudinal outcomes monitoring for safety and efficacy. In addition, there remain outstanding issues in this field preventing the broad clinical use of SC-β-cell therapy. These include improving scalability and dosing, eliminating the reliance on systemic immunosuppression in non-encapsulation strategies, and optimizing transplant site outcomes [163].

Another major challenge in the field remains in the generation of fully mature, glucose-responsive β-cells. While there are many protocols developed to differentiate SCs into immature, insulin-producing cells [68,69,70,71,72,73,74,75,76,77], there is difficulty in developing mature β-cells that dynamically secrete insulin in response to glucose to the degree of native human islets. Notably, transplantation of immature SC-β-cells promotes their functional maturation in vivo, suggesting key maturation signals are missing from current in vitro systems. *MAFA* expression is significantly upregulated following transplantation, consistent with its role in establishing functional β-cell maturity. Indeed, induction of *MAFA* expression has been shown to directly induce β-cell maturation from immature SCs or induce insulin production from non-β-cells [6,164].

Thus, strategies to increase *MAFA* expression in SC-β-cells, either through direct or indirect methods, are an important area of research. Current potential approaches include reducing oxidative stress, which contributes to post-translational MAFA loss during glucolipotoxicity [123], promoting chromatin states permissive for *MAFA* expression during β-cell maturation [165,166,167], enhancing circadian entrainment pathways which regulate *MAFA* rhythmic expression [75,160], or developing inducible systems to regulate the MAFA protein. While these strategies remain largely experimental, they may provide opportunities to restore endogenous MAFA activity under conditions of metabolic stress. New findings of rhythmic *MAFA* expression in mature SC-β-cells collectively point towards temporal and rhythmic regulation of *MAFA* expression for optimal SC-β-cell function [11,75]. These findings underscore the importance of better characterizing and precisely coordinating *MAFA* expression with developmental cues to optimize the generation of therapeutically relevant SC-β-cells.

## 8. Conclusions

Recent advances have transformed the view of MAFA from a β-cell maturation factor to a dynamically regulated integrator of developmental, metabolic, circadian, and stress-responsive signaling pathways. Collectively, studies from genetic mouse models, human donor islets, and SC-β-cells have revealed that MAFA activity must be maintained within a narrow functional range. Loss of *MAFA* impairs β-cell identity and GSIS, whereas stabilization of MAFA, as observed in MAFA-MODY, similarly disrupts β-cell function and glucose homeostasis. These findings support a model in which the timing, amplitude, and duration of MAFA activity are all critical determinants of β-cell health.

Despite significant advances, key questions remain. The upstream pathways governing MAFA induction during human β-cell maturation remain poorly defined, particularly given the substantial differences in developmental timing between rodents and humans. Likewise, recent evidence linking MAFA to circadian entrainment raises important questions regarding how rhythmic *MAFA* expression is established, maintained, and integrated with nutrient sensing and metabolic stress. Defining the transcriptional, epigenic, and environmental signals that coordinate these processes will be essential for understanding how mature β-cell identity is established and maintained.

Finally, emerging studies in SC-β-cells and circadian regulation of *MAFA* suggest that future therapeutic strategies will likely require restoration of physiological MAFA dynamics rather than simple enhancement of *MAFA* expression. Determining whether temporal *MAFA* expression and MAFA activity can be leveraged to improve β-cell replacement therapies, SC-β-cell maturation, or preservation of endogenous β-cell function represents an important challenge for the field. As efforts to develop therapies for diabetes advance, defining mechanisms that regulate MAFA abundance, activity, and rhythmicity will be critical to translating MAFA biology for therapeutic benefit.

## Figures and Tables

**Table 1:** Species-Specific Differences in β-cell MAFA Biology.

Feature	Rodent	Human	Implications for Disease Modeling
Developmental *MAFA* expression	First detected in the secondary transition of pancreatic development (E12.5–E13.5) and is restricted to insulin-producing cells [6,44,45]	Transcript not detected until second trimester and are lowly expressed throughout development [46,47,48], albeit no protein detected	Human *MAFA* expression is induced later than in rodents, suggesting species-specific developmental timing
Postnatal *MAFA* expression	Dominant *Maf* in β-cells postnatally and is highly expressed throughout adulthood [9,45,49]	*MAFA* is rarely detected in juvenile β-cells and increases later in childhood; robust expression is typically observed at ~10 years of age [48,50]	Human β-cells have at least two postnatal, glucose-responsive β-cell populations that differ by MAFA status
Relationship to β-cell maturation	Associated with acquisition and maintenance of β-cell identity and glucose-responsive insulin secretion [7]	Associated with mature β-cell identity and functional competence [51]	Suggests a conserved role for MAFA in β-cell maturation
*MAF* family expression in mature β-cells	Primarily *MafA*; *MafB* is expressed embryonically and restricted to α-cells postnatally [45]	Human β-cells can express *MAFA*, *MAFB*, or coexpress *MAFA/B* postnatally [51]	Human β-cells utilize distinct transcriptional networks not fully recapitulated in rodent models
MAFA in diabetes	Reduced in diabetic mouse models [9]	Reduced in donor islets and pathogenic variants cause MAFA-MODY [52,53,54,55]	Suggests a conserved role for MAFA dysfunction in diabetes pathogenesis

**Table 2:** Known β-cell MAFA target genes.

Pathway	Gene Name	Gene Function	Model Validated In	Reference
Glucose sensing	*Gck*	Catalyzes glucose to glucose-6-phosphate and regulates the rate of glycolysis and subsequent insulin secretion	Rodent	[95]
	*Slc2a2*	Encodes GLUT2, a glucose transporter essential for glucose uptake	Rodent	[95]
	*G6pc2*	Encodes an endoplasmic reticulum-resident glucose-6-phosphatase (G6Pase) subunit	Rodent	[96]
Insulin biosynthesis and processing	*Pcsk1*	Encodes Proprotein convertase subtilisin/Kexin1, converts proinsulin into active insulin	Rodent	[95]
	*Ins1*, *Ins2*	Insulin 1 (*Ins1*) and Insulin 2 (*Ins2*) are the two genes encoding insulin in mice	Rodent	[5,18,94,97,98]
	*INS*	Encodes human insulin peptide	Human	[5,18,94,97,98]
Granule formation	*Granuphilin*	Effector found on the membrane of insulin granules, aids in docking and fusion in exocytosis	Rodent	[49,99]
	*ZnT8*	Zinc transporter on insulin granules that influxes zinc from the cytoplasm	Rodent	[45]
Calcium dynamics	*CACNG4*	Encodes the calcium channel subunit gamma-4 (CaVγ4), an integral voltage-gated Ca^2+^ channel	Rodent and Human	[100]
	*Stim1*	Endoplasmic reticulum calcium sensor, helps to maintain Ca^2+^ homeostasis	Sheep	[101]
	*Ppp2ca*	Encodes the catalytic subunit or protein phosphatase 2A (PP2A), regulated by Ca^2+^ levels and is involved in a wide variety of processes	Sheep	[101]
	*Atp2a2*	Encodes the Sarco/endoplasmic reticulum Ca^2+^- ATPase (SERCA2) pump, which transfers Ca^2+^ from the cytosol into the ER lumen to maintain intracellular Ca^2+^ homeostasis	Rodent	[49]
Oxidative Phosphorylation	*Pc*	Encodes pyruvate carboxylase, plays a role in pyruvate metabolism, insulin secretion, and proliferation	Rodent	[95]
Proliferation	*Prlr*	Encodes prolactin receptor, essential for postnatal β-cell proliferation and adaptation to stress	Rodent	[102]
Exocytosis	*Stxbp1*	Encodes Syntaxin binding protein 1, regulates vesicle fusion	Rodent	[49,66]
	*STX1A*	Encodes a t-SNARE protein essential for granule docking, priming, and fusion	Rodent and Human	[66]
Essential identity genes	*Nkx6-1*	Plays a role in β-cell development, identity, and proliferation	Rodent	[95]
	*Pdx1*	Regulator of pancreatic development, β-cell maturation, and preservation of identity	Rodent	[95,103]
	*Neurod1*	Essential to the development and maintenance of the mature phenotype	Rodent	[95]
	*Ucn3*	Biomarker of functional and mature β-cells	Rodent	[7]
Other stimulant signaling	*Glp1r*	Encodes the glucagon-like-peptide (GLP-1) receptor and promotes proliferation and insulin biosynthesis	Rodent	[95]
	*PPP1R1A*	Encodes a subunit of protein phosphatase 1, involved in glycogen metabolism and other cellular signaling	Rodent and Human	[104]
	*ChrnB2*, *ChrnB4*	Encodes subunits of neuronal nicotinic acetylcholine receptors that regulate ion flow	Rodent	[105]
	*Adra2A*	Encodes A2A-adrenergic receptor, inhibits insulin secretion	Rodent	[105]
	*MaoB*	Encodes monoamine oxidase B and regulates intracellular monoamine levels needed for proper insulin secretion	Rodent	[106]
Circadian regulation	*Cry2*	Encodes Cryptochrome Circadian Regulator 2, a core transcriptional repressor of the circadian clock, regulates cyclic β-cell insulin secretion	Rodent	[107]
	*Per1*, *Per2*	Encodes Period Circadian Regulator 1/2, transcriptional repressors of the circadian clock, essential for postnatal proliferation and maturation	Rodent	[107]
	*Bhlhe40*	Encodes DEC1, a transcriptional modifier of the circadian clock and a regulator of β-cell maturation	Rodent	[107]

## Data Availability

No new data were created or analyzed in this study.
